# Overdose prevention for injection drug users: Lessons learned from naloxone training and distribution programs in New York City

**DOI:** 10.1186/1477-7517-4-3

**Published:** 2007-01-25

**Authors:** Tinka Markham Piper, Sasha Rudenstine, Sharon Stancliff, Susan Sherman, Vijay Nandi, Allan Clear, Sandro Galea

**Affiliations:** 1 Center for Urban Epidemiologic Studies, New York Academy of Medicine, New York, NY 10029, USA; 2 Harm Reduction Coalition, New York, NY 10001, USA; 3 Department of Epidemiology, Johns Hopkins Bloomberg School of Public Health, Baltimore MD 21205, USA; 4 Department of Epidemiology, University of Michigan School of Public Health, Ann Arbor, MI 48104, USA

## Abstract

**Background:**

Fatal heroin overdose is a significant cause of mortality for injection drug users (IDUs). Many of these deaths are preventable because opiate overdoses can be quickly and safely reversed through the injection of Naloxone [brand name Narcan], a prescription drug used to revive persons who have overdosed on heroin or other opioids. Currently, in several cities in the United States, drug users are being trained in naloxone administration and given naloxone for immediate and successful reversals of opiate overdoses. There has been very little formal description of the challenges faced in the development and implementation of large-scale IDU naloxone administration training and distribution programs and the lessons learned during this process.

**Methods:**

During a one year period, over 1,000 participants were trained in SKOOP (Skills and Knowledge on Opiate Prevention) and received a prescription for naloxone by a medical doctor on site at a syringe exchange program (SEP) in New York City. Participants in SKOOP were over the age of 18, current participants of SEPs, and current or former drug users. We present details about program design and lessons learned during the development and implementation of SKOOP. Lessons learned described in the manuscript are collectively articulated by the evaluators and implementers of the project.

**Results:**

There were six primary challenges and lessons learned in developing, implementing, and evaluating SKOOP. These include a) political climate surrounding naloxone distribution; b) extant prescription drug laws; c) initial low levels of recruitment into the program; d) development of participant appropriate training methodology; e) challenges in the design of a suitable formal evaluation; and f) evolution of program response to naloxone.

**Conclusion:**

Other naloxone distribution programs may anticipate similar challenges to SKOOP and we identify mechanisms to address them. Strategies include being flexible in program planning and implementation, developing evaluation instruments for feasibility and simplicity, and responding to and incorporating feedback from participants.

## Background

Fatal heroin overdose is a significant cause of mortality for injection drug users (IDUs) [1,2]. Half of all heroin drug users report at least one nonfatal overdose during their lifetime [3-5]. In New York City (NYC), an estimated 900 opiate users die from overdose each year, a figure that exceeds the number of deaths from homicide [6,7]. Many of these deaths are preventable because opiate overdoses can be quickly and safely reversed through the injection of Naloxone [brand name narcan], a prescription drug used to revive someone who has overdosed on heroin or other opioids. In addition, most overdoses occur in the presence of others, often drug-using peers [1,8] and many deaths may occur due to bystander response, mainly the reluctance of peers to contact emergency medical services for fear of police involvement [3].

Naloxone, an opiate antagonist, has long been administered by doctors and paramedics during emergency resuscitation after an opiate overdose. However, the provision and use of naloxone by drug users is a new and innovative approach to reducing opiate-related mortality. Currently, in several cities in the United States (US), drug users are being trained in naloxone administration and given naloxone for immediate reversals of opiate overdoses [9-13]. Preliminary reports from these programs have documented lifesaving events through peer administration without observed adverse effects [11,12,14] and increased overdose awareness and preparedness among opiate users in the programs. While the prevalence of opiate overdose mortality and the role of naloxone in opiate overdose prevention are gaining increased public health-related attention, there has been limited formal description of the development, implementation and evaluation of a large-scale naloxone administration training and distribution programs to IDUs and the lessons learned during this process [12,15].

In 2004, a pilot overdose prevention project at two NYC Syringe Exchange Programs (SEPs) trained approximately 100 drug users on overdose prevention. In 2005, the Harm Reduction Coalition (HRC), a national organization that uses education, interventions and community organizing to reduce drug-related morbidity and mortality, started the Skills and Knowledge on Overdose Prevention Project (SKOOP) [16]. SKOOP had three main goals: (i) to reduce overdose-related deaths through the distribution of naloxone hydrochloride to injection drug users in NYC; (ii) to build evidence for the effectiveness of take-home naloxone in harm reduction settings, and (iii) to create wider support for the inclusion of naloxone in harm reduction, methadone, and other public health programs.

The NYC HRC, NYC SEPs, the New York Academy of Medicine, and the Johns Hopkins Bloomberg School of Public Health collaborated on program design, implementation and evaluation of SKOOP at NYC SEPs. In this manuscript, the collaborators will discuss challenges and lessons learned from the implementation of SKOOP and offer recommendations for other SEPs that are considering the distribution of naloxone to drug users as a health promotion and disease prevention initiative.

## Methods

### Program design

**Figure 1:**
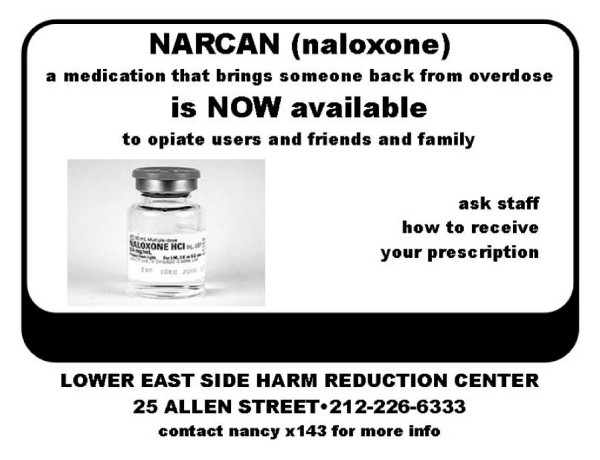
Recruitment flyer used in SKOOP program by staff at Syringe Exchange Programs in New York City.

**Figure 2:**
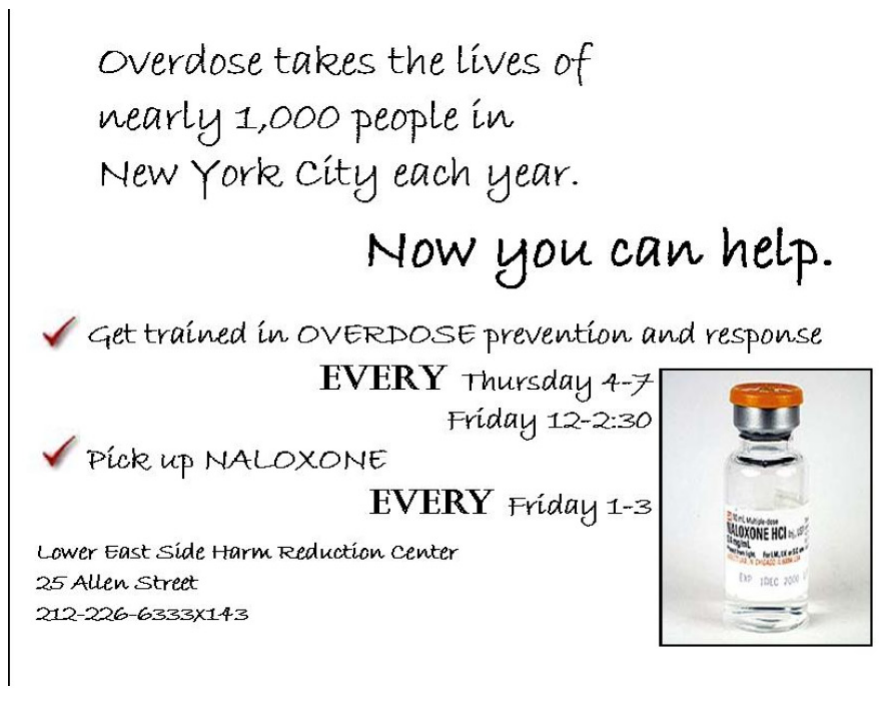
Recruitment flyer used in SKOOP program by staff at Syringe Exchange Programs in New York City.

Between March 2005 and March 2006, 1004 IDUs were trained in overdose prevention and given a prescription for naloxone. Participants were over the age of 18, current participants of NYC SEPs, and current or former drug users. Outreach efforts to recruit participants into the program, included flyers, word of mouth, enlistment during syringe exchange sessions and through other educational and medical programs. Figure 1 and Figure 2 show examples of recruitment flyers used in the program.

### Overdose prevention and reversal curriculum

All participants received an overdose prevention training by SEP staff who were trained by the onsite medical director and physician. The overdose training course, SKOOP, was modeled after existing naloxone distribution programs in Chicago and San Francisco [10,17] and is available from the authors. Opiate users were trained either individually, in pairs, or in small groups (5–15 people) by SEP and HRC staff. Each SKOOP training ranged from 10–30 minutes. The SKOOP curriculum focused on overdose prevention education and naloxone administration. Overdose prevention training included teaching and discussing (a) the causes of opiate overdose (i.e. loss of tolerance, mixing drugs, physical health and variation in strength of 'street drugs'), (b) how to avoid an opiate overdose (i.e. know your tolerance and supply, control your high, injection techniques, aware of risks of mixing drugs, and minimize using alone), and (c) signs of an opiate overdose. The naloxone training included information on naloxone, education about appropriate responses to opiate overdose (i.e. calling 911 and performing rescue breathing) and instructions on naloxone administration (intramuscular injection practices, the use of naloxone only with opiate-related overdose and the potential need for a second dose of naloxone). In some trainings, a rescue dummy was available to demonstrate and practice rescue breathing procedures; in all trainings, the correct use of a rescue breathing mask was modeled to participants. SEP trainers also discussed methods of cooperating with police and medical staff post-naloxone administration and the importance of talking to drug using partners about naloxone and overdose response.

### Naloxone kit and naloxone prescriptions

**Figure 3:**
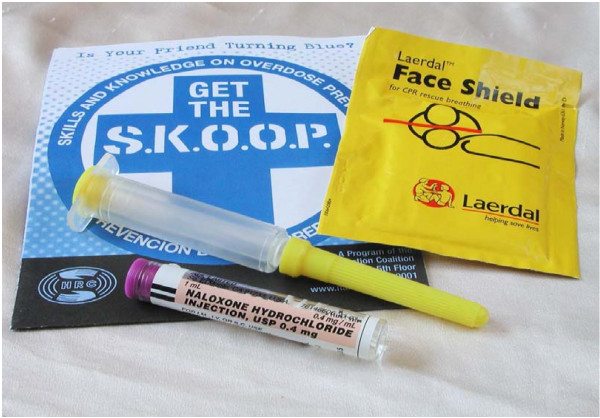
**The naloxone kit provided to participants in the SKOOP program**. Photo credit: Harry Peronius

Upon successful completion of the overdose prevention training, participants in the program met with an on-site physician for a brief (1–2 minutes), targeted medical history who then gave each participant a "naloxone kit," a carrying case with the following contents: two doses of naloxone in pre-filled syringes (1 mg/ml), a rescue breathing mask, and written information summarizing overdose revival steps. A prescription was also give as proof of the legitimacy of the medication. The medical history was also an opportunity for the medical doctor to inquire if participants needed referrals for primary care or drug treatment and to ensure that the participant understood the material delivered during the SKOOP training. Refills were given as needed by the medical doctor. Figure 3 contains a picture of the contents of the naloxone kit.

## Results and Discussion

### Challenges and Lessons Learned

We will describe here both challenges and lessons learned from SKOOP and conclude with recommendations for future naloxone distribution programs. These insights are collectively articulated, discussed and summarized by both the evaluators and implementers on the project. This diverse team included the medical director on site at the SEPs who provided SKOOP training for SEP staff, prescriptions and medical histories for participants, and liaison with the external evaluators; external evaluators (both quantitative and qualitative-focused) who assisted with study and survey design and implementation; project managers who trained and supervised survey interviewers and coordinated the overall programmatic efforts; and the administrator at Harm Reduction Coalition who had overall responsibility for project implementation. Together, the team identified six primary challenges and lessons learned from SKOOP, including: a) political climate of naloxone distribution b) extant prescription drug laws; c) initial low levels of recruitment into the program; d) training methodology modifications; e) conducting a formal evaluation; and f) evolution of program response to naloxone.

#### a. Political climate of naloxone distribution

Proponents of naloxone administration programs define opiate overdose as a health promotion, disease prevention, and harm reduction issue. Opponents see naloxone administration programs as vehicles to encourage drug use among opiate users who will view naloxone as an "escape valve" or "safety net" and an opportunity to increase their drug use issue [12,18]. For these reasons, naloxone provision to drug users is a politically charged issue. Changes in the New York State legislature regarding overdose prevention coupled with a growing awareness of overdose prevalence mortality assisted the program's feasibility. In April 2006, a New York State law regarding opioid overdose prevention now authorizes the state health commissioner to establish standards for overdose prevention programs and the use of naloxone by non-medical staff in the case of an overdose [19]. This law was unanimously passed in the House and Senate and supported by the Medical Society of the State of New York.

Overdose prevention is attractive to many agencies that provide services to drug users and yet have not chosen to furnish syringes for their clients. Therefore, the SKOOP project has been educating many agencies including drug treatment providers, housing agencies and primary care providers on overdose prevention and naloxone administration. Two methadone providers and one primary care unit are preparing to start providing naloxone, and a dozen other agencies are actively interested in providing these services [20]. Overdose is increasingly becoming recognized as a serious public health issue and naloxone is increasingly seen as a new and innovative approach to reducing opiate-related mortality.

#### b) Extant prescription drug laws

In most states, and until recently in New York, it was illegal for someone with a prescription medicine to administer it to someone for whom it was not prescribed. This, for example, would apply to naloxone (for a drug-using partner or an unknown overdosing person) or an extra dose of Azithromycin (for the unseen partner of a patient being treated for Chlamydia). The recent New York State opioid overdose prevention law (April 2006) that allows for non-medical persons to administer naloxone has facilitated an opportunity for a range of organizations to implement overdose prevention programs. New Mexico has similar legislation [21] and Connecticut's law allows for licensed health care practitioners to prescribe, dispense or administer naloxone to drug users to prevent overdose deaths without civil or criminal liability [22]. However even in those states, naloxone remains a prescription medication with many states requiring a face-to-face encounter for a medication to be legally prescribed. Current prescription drug laws provide a challenge in finding and recruiting medical physicians on naloxone distribution programs. To facilitate and expedite medical involvement in the project, SKOOP has created a brief targeted medical record, accomplished in one minute for most SKOOP participants, and the prescriber/medical physician does not need to participate in the training. The SKOOP project is also currently working with medical staff in contact with drug users both at syringe exchanges and drug treatment programs. In addition, pharmacies have begun to show strong interest in stocking and providing naloxone [23].

#### c. Initial low levels of recruitment into the program

In the early months of the program, participants' misconceptions about naloxone were initially seen as a barrier. Some participants who had negative perceptions of naloxone from street lore or personal experience were initially unwilling to participate in the program [24]. Primarily, participants were concerned with the dopesickness–or opiate withdrawal characterized by shaking, headache, nausea, and vomiting–associated with using naloxone. SEP staff shifted to generalizing messages to increase program involvement with a focus on "overdose prevention" and "saving lives." Outreach methods and flyers were modified to emphasize health promotion and overdose prevention to encourage opiate users to partake in the training and learn about the benefits of naloxone. Additional efforts to change perceptions included conducting two focus groups among 13 participants to receive feedback about the program and better understand participant experiences and views on naloxone [24].

#### d. Training methodology modifications

Prior to SKOOP, a pilot project with 25 IDUs was conducted [11] and trainings were provided once a month using a pair model, in which drug users were required to present with a drug using partner as this allows for each participant to theoretically be injected with their own naloxone. Participants reported difficulties finding a "pair" and coordinating schedules with their pair. It was soon recognized that a lower threshold, "drop in" approach, that targeted individuals was most appropriate to facilitate participant needs. SKOOP learned from the pilot experience and agencies have since developed many models for the trainings and some employ multiple methods. Trainings may be in the storefront or conducted during outreach. They may be one-on-one, or in groups and can last between 10 and 30 minutes depending on the needs of the group. While naloxone administration is a life-saving intervention, it is also very simple to administer. Some of the highest risk users may not have more than 10 minutes to spend in a training as they are trying to sustain a heroin habit or may not have a long attention span because they are under the influence of several drugs. Trainings, therefore, were tailored to be quick, instructive and tailored to the needs and time of each group. While some participants were trained in drop-in settings, the largest numbers of users were trained on street corners. To illustrate this point, one day in February, the SEP training team was set up at a new site in a warm room with coffee and donuts. No one came. The team then reassembled in a park near a methadone program and 26 participants were trained. The process of selecting trainers changed in some agencies during program implementation. Initially many SEPs felt that senior staff needed to facilitate the trainings which presented some conflict as these staff members have responsibilities away from the front lines. As knowledge and comfort with the program developed, it became clear that outreach workers and peer educators were not only capable of conducting the trainings, but that their skills were well-suited to the tasks.

#### e. Conducting a formal evaluation

Achieving a balance between research/evaluation needs and program/participant needs is an ongoing process that requires effective management and communication, including collaboration in the development of processes for study design, implementation and dissemination of findings. A pilot with 25 IDUs at a SEP in Lower Manhattan was conducted in the early months of the program to identify project feasibility [11]. Evaluation methods from the pilot included a baseline and three month follow-up instrument to assess prior overdose experience and responses to witnessed overdoses, including naloxone experience. In addition, during the pilot, a pre-post training test was administered to measure participant knowledge of naloxone knowledge and administration techniques. Although we were able to conduct a small scale pilot and follow-up effectively, there were several challenges that quickly became apparent in formally evaluating this large-scale program. Scientifically, a longitudinal evaluation that would enable the tracking of participants to follow experiences of participants from initial entry into the program to experiences with naloxone administration and overdose prevention would be desirable. There were however, several barriers to the implementation of such a longitudinal study. First, confidentiality issues were paramount for the SEPs, and as a result these obviated follow-up with participants in the broader pilot. Second, it was determined that program resources for the purposes of follow-up tracking were insufficient and as such, it would require substantial SEP staff effort to properly conduct follow-up; such effort was not realistic given the already over-extended time effort of SEP staff. Therefore, for this evaluation, we settled on a simple post-design evaluation to collect data on overdose and naloxone experience among those participants who return for a naloxone syringe refill. We would suggest that a longitudinal study that is separately funded from the naloxone distribution effort and as such resourced to conduct careful, anonymized follow-up of drug users who receive naloxone, is needed to provide data about the long-term consequences of naloxone dispensing.

#### f. Evolution of program response to naloxone

Despite initial concerns by participants about the effects of naloxone (i.e. opiate withdrawal), there was a positive response to naloxone in the NYC program. During the program, it quickly became evident that the availability of naloxone was not just important to IDUs who are at risk of overdose and rely on naloxone to save their own life or the life of a friend, but also to the people who live in the communities where injection drug use is prevalent or to service providers of IDUs. The significance of naloxone was apparent in four different ways. First, SEP staff overwhelmingly supported the distribution of naloxone and were eager both to carry naloxone and to participate in the program as trainers. Second, SEP participants strongly desired the opportunity to have take-home naloxone and the program continues to increase in size and demand. Third, many participants expressed anger or frustration that their friend died of an overdose because naloxone was not available sooner. And finally, a few weeks after naloxone became available and popular among the IDU population in Lower Manhattan, a few individuals whose work (i.e. soup kitchen) brought them in contact with IDUs attended a training to receive naloxone to keep at work in the event of an overdose.

## Conclusions and recommendations

Naloxone distribution programs in the US are ongoing in Chicago, Baltimore, San Francisco, New Mexico and New York City. Additional community-based organizations interested in minimizing the adverse consequences of drug use in several cities in the US, including Los Angeles, Providence, Pittsburgh and Boston, are in the process of planning and developing naloxone administration programs for drug users. The recommendations presented here are designed to assist other SEPs and health promotion centers in their planning, implementation and evaluation of similar programs for opiate users. We recognize that this is not a formal process evaluation but given the innovative nature of the project and the unusual collaborative and evaluative processes, we feel that there is valuable insight to be gained from the team's experiences with naloxone distribution in NYC over the past two years.

First, take-home naloxone distribution programs for opiate users are feasible and both programmatic experience and data suggests that drug users can be trained to respond to heroin overdose by giving naloxone. The Chicago Recovery Alliance (CRA) has operated one of the largest naloxone distribution programs to date. Since January of 2001, CRA has reported equipping approximately 3,500 people with naloxone, resulting in 319 reversals which were associated with 20% decrease in overdoses in 2001 and a 10% decrease in 2002 and 2003. This reversed a steady increase in heroin overdoses since 1991 [25]. In Baltimore, naloxone distribution began in April 2004; as of March 2006, 951 individuals have been trained in naloxone administration and a reported 131 overdoses have been reversed with the use of naloxone [14]. Since December 2003, through a collaboration between Project DOPE and the San Francisco Department of Public Health, 700 participants have received a prescription for naloxone with 170 reported overdoses reversed with naloxone [10]. Currently in New York City, since April 2005, 1485 people have been trained and received naloxone prescriptions with approximately 104 reported overdose reversals [20]. We caution that all such program evaluations need to be understood within the context of the outcome they are evaluating.

Second, flexibility is essential in the development, implementation and evaluation of naloxone administration programs. This flexibility means adapting overdose prevention training curriculum to be delivered quickly and effectively in numerous settings– whether in a designated room at a SEP with a few participants, or outside in an often chaotic public space during needle exchange sessions. In addition, each SEP requires adaptation of program components to fit participant needs and experiences. A SEP that works with runaway or homeless adolescents considers different programmatic needs and issues than a SEP that attracts an older drug-using population.

Third, evaluation components should be designed for feasibility and simplicity. A brief assessment that can be administered in a few minutes to participants who may be high or unresponsive or at first unwilling to participate is more practical than a detailed questionnaire.

Fourth, the program is entirely dependent on opiate user participation–responding to and incorporating feedback from participants (i.e. multiple outreach strategies, flexible hours for naloxone prescription by the medical physician, an abbreviated training curriculum) is integral for program success.

We recommend that additional cities in the US initiate take-home naloxone programs for drug users because they are feasible and effective; we urge further assessments of new data on participant experience with naloxone and overdose prevention; and we recommend additional systematic evaluations with follow up components. In NYC, drug users at local SEPs continue to be trained in overdose prevention and naloxone administration, an initiative that may be instrumental in reducing overdose mortality in NYC.

## Competing interests

The author(s) declare that they have no competing interests.

## Authors' contributions

TMP coordinated the study and drafted the manuscript. SR coordinated the study. SS provided supervision and coordination at the SEPs and performed trainings and provided naloxone prescriptions. SGS participated in the conception and design of the study. VN performed the statistical analysis. AC participated in the design of the study and obtained funding for the study. SG conceived of the study, helped to obtain funding, participated in its design and coordination and helped to draft the manuscript. All authors read, critically edited, and approved the final manuscript.
